# Supplementary material for: Crystal structure of the human PRPK–TPRKB complex
Source: Commun Biol. 2021 Feb 5;4:167. doi: 10.1038/s42003-021-01683-4 (PMC7864929; doi:10.1038/s42003-021-01683-4)
Supplement: Supplementary file 2 — Supplementary Information [file 42003_2021_1683_MOESM2_ESM.pdf]

Supplementary Fig. S1

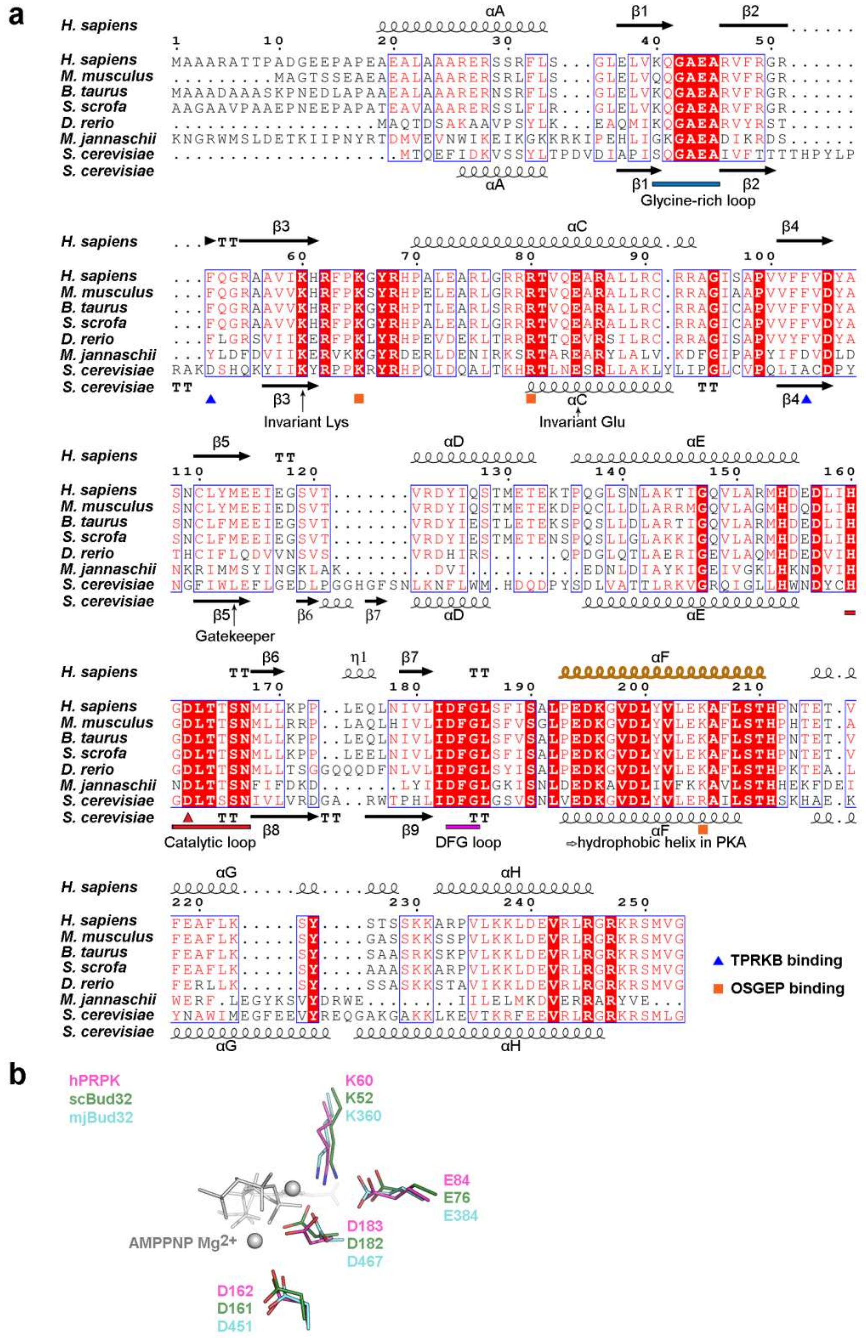

Supplementary Fig. S1: PRPK multiple sequence alignment and K/E/D/D structural alignment.

(a) PRPK amino acid sequences from human (Uniprot Q96S44), mouse (Uniprot Q99PW4), bovine (Uniprot A5PK80), pig (Uniprot F1SBF6), zebrafish (Uniprot F1QB90), *M. jannaschii* (Uniprot Q58530)

and yeast (P53323) were aligned using Clustal Omega and displayed with ESPript 3. Secondary structure of PRPK observed in our model and in yeast Bud32 structure (PDB ID 4WW5) is displayed on top and bottom of the sequence, respectively. Conserved kinase elements are indicated. Key residues later found to be important for TPRKB binding, and OSGEP binding are labeled with blue triangles, and orange squares, respectively.

(b) The K/E/D/D signature motif (K60, E84, D162, and D183 in PRPK) is extremely conserved at the structural level. Human PRPK, yeast and archaeal Bud32 residues are colored magenta, green and cyan, respectively.

Supplementary Fig. S2

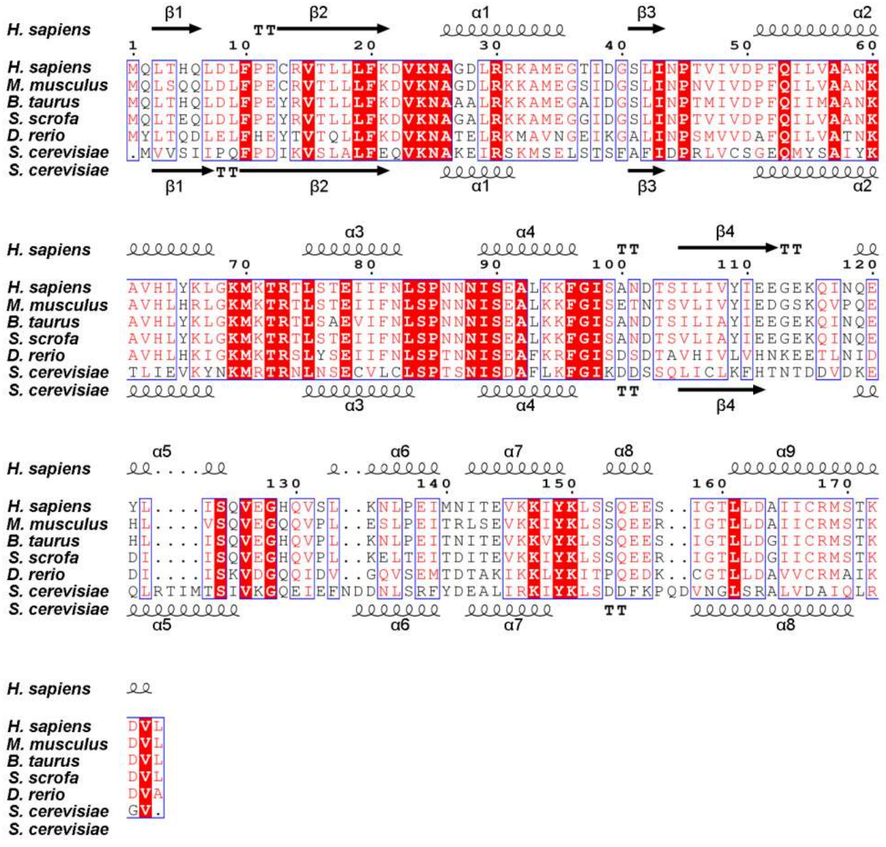

Supplementary Fig. S2: TPRKB multiple sequence alignment.

TPRKB amino acid sequences from human (Uniprot Q9Y3C4), mouse (Uniprot Q8QZZ7), bovine (Uniprot E1BN75), pig (Uniprot B8Y649), zebrafish (Uniprot F1QZ15), and yeast (Q03705) were aligned using Clustal Omega and displayed with ESPript 3. Secondary structure of TPRKB observed in our model and in yeast Cgi121 structure (PDB ID 4WW5) is displayed on top and bottom of the sequence, respectively.

Supplementary Fig. S3

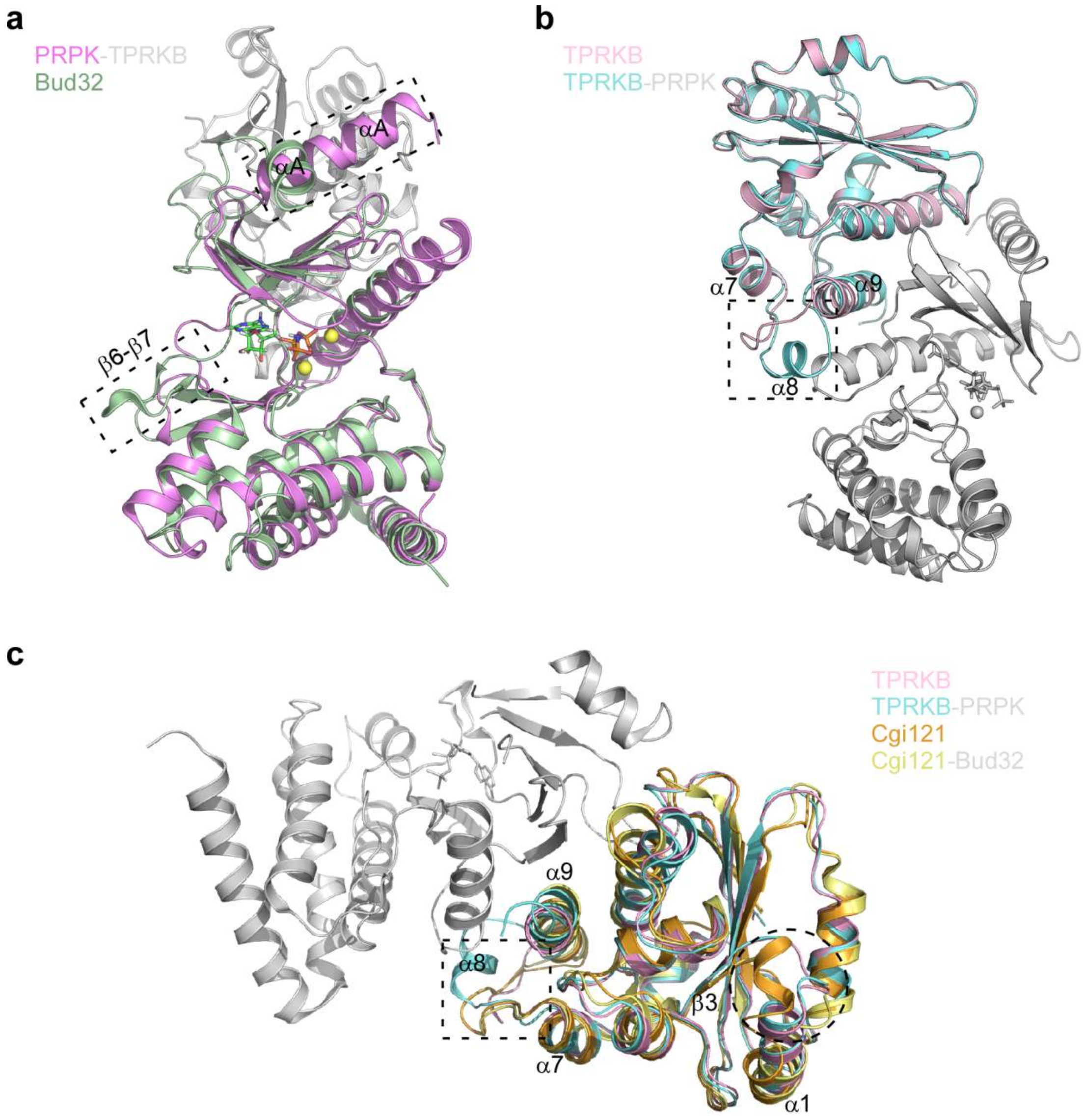

Supplementary Fig. S3: Structural comparison.

(a) The human PRPK structure is different from yeast Bud32 in two areas (dashed squares). First, helix αA adopts a completely different orientation in the two structures. PRPK helix αA is longer and both ends of the helix contact TPRKB. Second, the hinge region of Bud32 forms an extra two strand β-sheet (β6-β7), while the hinge region of PRPK is shorter. The Bud32 structure is also in a complex with Cgi121, but for clarity, the Cgi121 portion is not shown.

(b) Upon forming a complex with PRPK, a segment of loop in TPRKB is reorganized into a helix (α8) and moves towards PRPK (dashed square). TPRKB in the complex structure is colored aquamarine.

(c) The α8 structural change is not observed in yeast Cgi121 (dashed square), and the strucutral change in Cgi121 upon complexation with Bud32 (dashed circle) also does not occur in human protein.

Supplementary Fig. S4

a

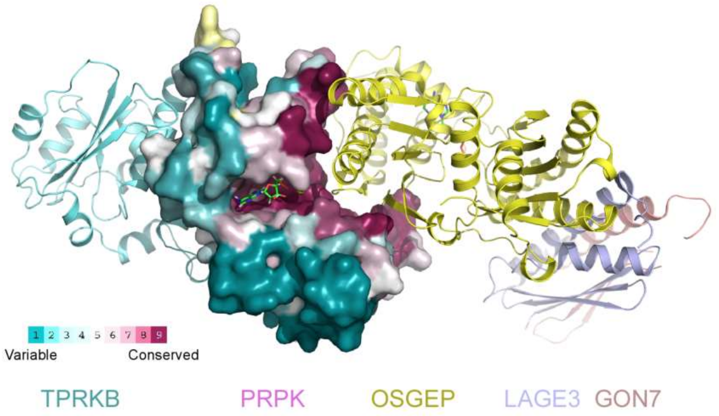

b

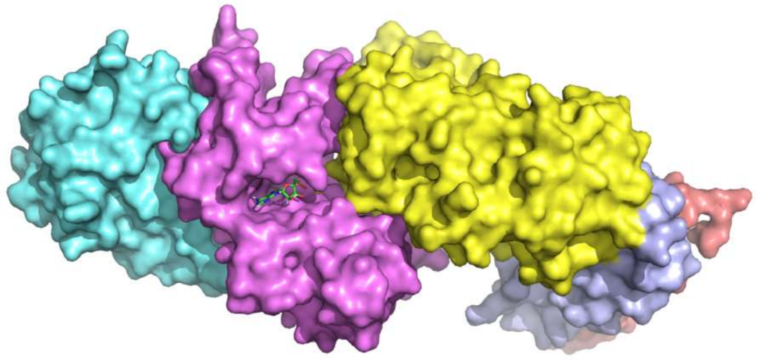

Supplementary Fig. S4: A model of the human EKC/KEOPS complex.

(a) A ConSurf style surface representation of PRPK in the context of the EKC/KEOPS complex. The OSGEP binding site, as well as the ATP binding pocket, on PRPK is highly conserved.

(b) Surface representation of the whole EKC/KEOPS complex. The AMPPNP in the PRPK ATP binding pocket is shown as stick. The conventional kinase substrate binding site is blocked by OSGEP.

Supplementary Fig. S5

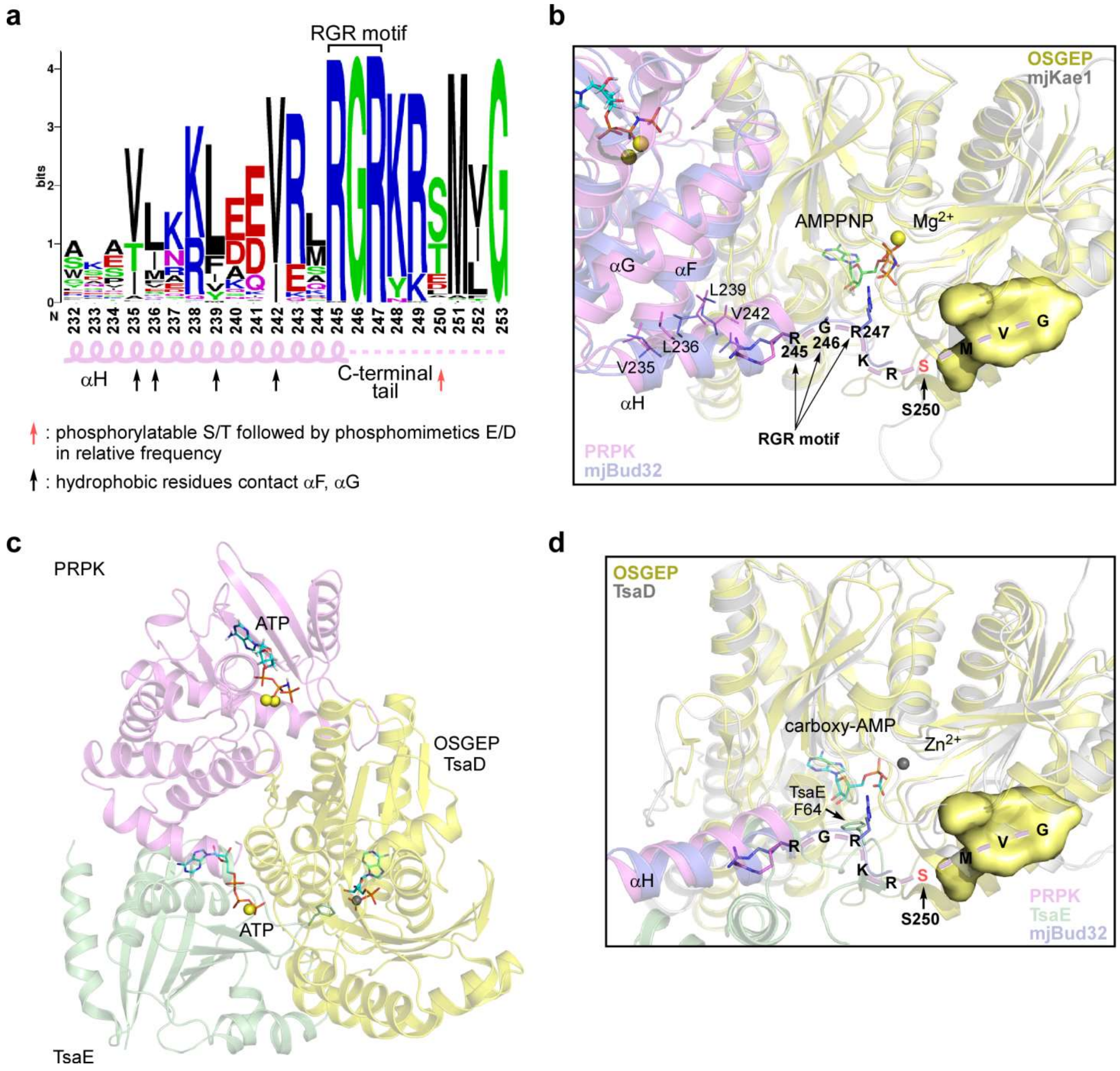

Supplementary Fig. S5: A potential regulatory role of the conserved PRPK C-terminal tail.

(a) Sequence logo of the PRPK helix αH and the C-terminal tail.

(b) The human PRPK and OSGEP structure is aligned to the corresponding part of the mjBud32-Kae1 crystal structure. The residues of helix αH involved in the inter-helix hydrophobic interaction are conserved at the primary sequence level (A), and align well in the structure (PRPK V235, L236, L239, and V242, along with the corresponding residues in mjBud32, are shown as stick). The first arginine of

the RGR motif also matches well in the aligned structure (PRPK R245 and the corresponding residues in mjBud32 are shown as stick). Based on structural conservation, we propose the PRPK C-terminal tail in the PRPK-OSGEP structure could follow a similar trajectory as the mjBud32 tail in the mjBud32-Kae1 structure. Thus, PRPK R247, the second arginine of the RGR motif, may also reach into the OSGEP catalytic center, as seen in the mjBud32-Kae1 structure (blue stick). This potentially would interfere with substrate binding and play an inhibitory role. Continuing to follow the mjBud32 tail in the crystal structure, this would place PRPK S250 close to, and PRPK M251-V252 right on top of a hydrophobic surface on OSGEP (surface representation in yellow).

(c) Similarity between human and bacterial t6A modification complex. The human PRPK-OSGEP model is aligned to the *T. maritima* TsaE-TsaD structure (tmTsaE-TsaD, PDB ID 6N9A), by matching the OSGEP to the tmTsaD structure (32% identity). For clarity, tmTsaD is not shown. Note that, OSGEP/TsaD both collaborate with an ATPase subunit. In eukaryotes, it is PRPK and in bacteria, it is TsaE.

(d) A loop in TsaE occupies the TsaD catalytic pocket, with TsaE F64 deeply inserted (green stick). This blocks tRNA substrate binding. Thus, a similar regulatory mechanism may exist in both the bacterial and eukaryotic complexes. The PRPK-OSGEP model, mjBud32-Kae1 structure, and tmTsaD-TsaE structure are aligned, according to the OSGEP/Kae1/TsaD portion. For clarity, mjKae1 is not shown and PRPK/mjBud32/tmTsaE only shows the region of interest.

Supplementary Fig. S6

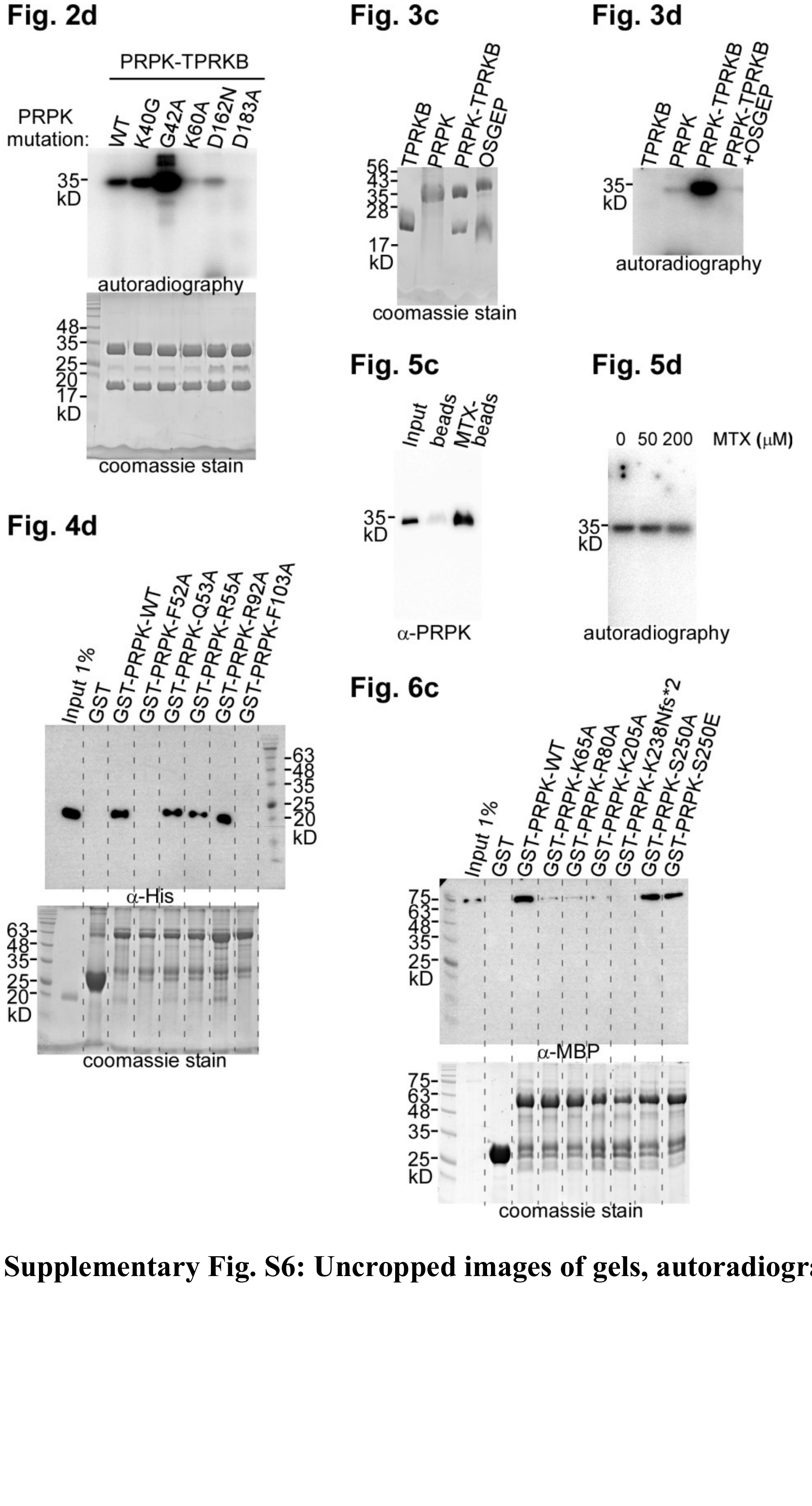

Supplementary Fig. S6: Uncropped images of gels, autoradiographs and blots.
